# The interaction of lipocalin-2 and astrocytes in neuroinflammation: mechanisms and therapeutic application

**DOI:** 10.3389/fimmu.2024.1358719

**Published:** 2024-03-12

**Authors:** Qianqian Tan, Chenxi Zhang, Xiuqin Rao, Wei Wan, Wei Lin, Shupeng Huang, Jun Ying, Yue Lin, Fuzhou Hua

**Affiliations:** ^1^ Department of Anesthesiology, the Second Affiliated Hospital of Nanchang University, Nanchang, Jiangxi, China; ^2^ Key Laboratory of Anesthesiology of Jiangxi Province, Department of Anesthesiology, The Second Affiliated Hospital of Nanchang University, Nanchang, Jiangxi, China; ^3^ Department of Anesthesiology, The First Affiliated Hospital of Gannan Medical University, Ganzhou, Jiangxi, China

**Keywords:** Lcn-2, astrocytes, neuroinflammation, NF-κB signal pathway, therapeutic application

## Abstract

Neuroinflammation is a common pathological process in various neurological disorders, including stroke, Alzheimer’s disease, Parkinson’s disease, and others. It involves the activation of glial cells, particularly astrocytes, and the release of inflammatory mediators. Lipocalin-2 (Lcn-2) is a secretory protein mainly secreted by activated astrocytes, which can affect neuroinflammation through various pathways. It can also act as a pro-inflammatory factor by modulating astrocyte activation and polarization through different signaling pathways, such as NF-κB, and JAK-STAT, amplifying the inflammatory response and aggravating neural injury. Consequently, Lcn-2 and astrocytes may be potential therapeutic targets for neuroinflammation and related diseases. This review summarizes the current knowledge on the role mechanisms, interactions, and therapeutic implications of Lcn-2 and astrocytes in neuroinflammation.

## 1 Introduction

Neuroinflammation is the brain’s immune response to various harmful stimuli, including infection, injury or neurodegenerative disease (1), which is primarily mediated by brain-resident microglia and astrocytes (2), along with some periphery infiltrating immune cells (3), involving the activation of neuroglia and the up-regulation of inflammatory mediators (4). While neuroinflammation can act as a defense mechanism to protect the nervous system by removing cell debris and promoting tissue repair, persistent inflammation can impede recovery and have detrimental effects (5, 6). Numerous studies have shown that neuroinflammation is an important component of the pathological processes of various neurodegenerative diseases and brain injuries, such as Alzheimer’s disease (AD), Parkinson’s disease (PD), and multiple sclerosis (MS) (1, 7). Currently, the detection and treatment of neuroinflammation present significant challenges, necessitating further basic and clinical research to elucidate the molecular mechanisms, developmental processes, and intervention strategies associated with neuroinflammation.

Astrocytes, the most abundant cells in the central nervous system (CNS) (8), play a pivotal role in maintaining neuronal function, regulating the blood-brain barrier (BBB), controlling ion concentration, supporting neuronal nutrition and metabolism, and participating in immune response (9–15). During neuroinflammation, astrocytes are activated into reactive astrocytes, which exhibit different phenotypes and functions (16, 17). Reactive astrocytes can secrete various cytokines and chemokines, promoting immune cell infiltration, thereby intricately influencing CNS inflammation (18, 19). Previous studies have indicated that Lipocalin-2 (Lcn-2) is overexpressed and assumes a crucial role in various pathological conditions related to neuroinflammation, including brain injury, stroke, AD and others (20). And it is primarily secreted by activated astrocytes (21). Studies suggest that Lcn-2 can modulate the astrocyte reactivity, disrupt the BBB (22), induce neuronal death, and inflammation, potentially contributing to neurodegeneration (23). Therefore, Lcn-2 and astrocytes hold significant biological and clinical relevance in neuroinflammation, with Lcn-2 secreted by astrocytes potentially serving as a therapeutic target for various brain diseases with neuroinflammatory features.

This review will focus on the mechanisms, interactions, and effects of neuroinflammation, as well as the therapeutic methods and significance of Lcn-2 and astrocytes as targets for neuroinflammation treatment.

## 2 The molecular structure and biological function of Lcn-2

Lcn-2 is a 25KDa circulating protein from the lipocalin superfamily (24). The discovery of Lcn-2 can be traced back to 1989 when scientists identified a novel protein by its messenger RNA 24p3 in mouse model kidney cells infected with simian virus 40 (SV-40) (25). Subsequently, Lcn-2 was isolated from human neutrophil granules and mouse kidney cells released during infection and inflammation (26, 27). It is also called neutrophil gelatinase-associated lipocalin (NGAL), siderocalin and uterocalin (27, 28). The structure of Lcn-2 consists of an α-helix and an eight-stranded antiparallel β-barrel connected by a disulfide bond between Cys-78 and Cys-177 on both sides of the molecule (29). Lcn-2 possesses a funnel-shaped binding pocket allowing it to transport small or hydrophobic molecules, such as lipids, steroid hormones, and iron ions (30, 31).

In normal tissues, Lcn-2 is restricted to peripheral organs, such as the kidney, liver, bone marrow and adipose tissue. However, in organs like the brain, heart, skeletal muscle and spleen, Lcn-2 is only expressed under pathological states, such as infection, inflammation and cancer (28, 32). As an acute-phase protein, Lcn-2 is mainly secreted by astrocytes in various CNS diseases, with its expression generally elevated in response to inflammatory and pathological stimuli (21, 33). Moreover, neurons, endothelial cells, microglia and infiltrating neutrophils can also up-regulate Lcn-2 in the disease context (20, 34, 35).

Lcn-2 can bind to the cell surface receptors megalin and 24p3R [also known as LCN2R, solute carrier SLC22A17 or brain-type organic cation transporter (BOCT)] (36). Megalin is mainly expressed on the apical surface of epithelial cells, including renal proximal tubule, epididymal and thyroid cells, and is also found in reactive astrocytes and neurons (37), while 24p3R is highly expressed in astrocytes, neurons, microglia, endothelial cells, neutrophils, macrophages, kidney epithelial cells, epithelia of respiratory and alimentary tracts (20, 32, 38). Megalin-internalized Lcn-2 is mainly involved in cellular iron hemostasis (39), while binding to 24p3R mainly mediates cell death and iron uptake (40). Additionally, studies have shown that bone-derived Lcn-2 can cross the BBB and bind to MC4R in the hypothalamus, thereby inhibiting the neural signals of appetite and food intake, and regulating energy metabolism and blood glucose levels.

As individuals age, the expression of Lcn-2 increases throughout the systemic and the CNS. Studies have shown that Lcn-2 regulates various neurobiological processes, including inflammation, cell death/survival signaling, iron metabolism, BBB disruption and others, influencing the pathophysiology of age-related brain diseases (20, 41). Long-term increases in Lcn-2 levels can make the brain more susceptible to various age-related brain diseases, potentially contributing to their development (42).

Lcn-2 is a multifunctional protein involved in inflammation regulation, infection defense, iron homeostasis, and cell migration and differentiation, among other physiological processes (20, 41, 43). It has been found that specific overexpression of Lcn-2 in astrocytes within the hippocampal region can lead to neuroinflammation and cognitive impairments (7). A spinal cord injury (SCI) study demonstrated that Lcn-2 exhibits pro-inflammatory properties, while mice with the Lcn-2 gene knocked out significantly suppress the formation of NLRP3 inflammasomes and inflammatory response. Additionally, Lcn2 can mediate the input and output of intracellular iron to regulate iron homeostasis by binding to 24p3R. A study has revealed that Lcn-2 can induce the expression of pro-apoptotic protein Bim by decreasing intracellular iron levels, thus leading to apoptosis (40). Furthermore, several drugs have been shown to modulate Lcn-2 levels or activity (42). For instance, researchers found that inhibiting Lcn-2 expression using Sailuotong capsule effectively prevented neuroinflammation and recognition memory deficits induced by cerebral ischemia (44). These findings illustrate the critical role of Lcn-2 in neuroinflammatory processes and suggest the potential for pharmacological interventions targeting Lcn-2.

## 3 The signaling pathways and mechanisms of Lcn-2 in the regulation of neuroinflammation

As an inflammatory protein, Lcn-2 is abundantly released under inflammatory stimuli and employs different signaling pathways and mechanisms to modulate inflammation levels. The following will introduce some of them.

### 3.1 NF-kB signaling pathway

One of the signaling pathways is Nuclear factor-kappa B (NF-κB), which is important for inflammation regulation (33). NF-κB can be activated by various stimuli and enter the nucleus to initiate the expression of a series of inflammation-related genes. Studies have shown that Lcn-2 can trigger NF-κB phosphorylation by binding to cell membrane receptors (24p3R), then initiate the expression of inflammatory genes such as interleukin (IL)-β, IL-6 and tumor necrosis factor (TNF)-α (20). Furthermore, NF-κB can enhance the expression of Lcn-2 by binding to its gene (45), creating a positive feedback loop that amplifies inflammation.

### 3.2 JAK-STAT signaling pathway

Another signaling pathway is the Janus kinase-signal transducer and transcription activator (JAK-STAT) (46). Lcn-2 can activate the JAK-STAT pathway, which is secreted by activated microglia and astrocytes, leading to increased inflammatory cells and factors, resulting in neuronal damage and aggravating neuroinflammation. In addition, Wang et al. found evidence of Lcn-2/JAK2-STAT3 crosstalk contributing to the activation of neurotoxic microglia and astrocytes in the spinal cord after SCI, and inhibition of this crosstalk can reduce neuroinflammation and promote tissue repair (47).

### 3.3 Immune cell activation

By releasing cytokines that can be pro-inflammatory or anti-inflammatory, and by phagocytosing or clearing harmful substances, immune cells can combat infection and injury, which affects neuroinflammation (48). Lcn-2 can influence the activation state of immune cells by binding to 24p3R and modulating the production and release of cytokines, communication molecules between cells. For instance, Lcn-2 can make macrophages secrete more TNF-α and IL-β, which are harmful cytokines that promote neuroinflammation (40). Additionally, in a mouse model of MS, Sciarretta et al. identified that dysfunctional adipocytes can release Lcn-2, activate innate immunity, and shape the pro-inflammatory macrophage phenotype. Genetic deficiency of Lcn-2 can reduce inflammatory macrophage infiltration in the spinal cord, highlighting the role of Lcn-2 in exacerbating inflammation through immune cell activation in MS (49).

### 3.4 Cell apoptosis and survival

Apoptosis is an ordered and controllable cell death process that maintains tissue homeostasis and clears damaged cells. However, excessive or inappropriate cell apoptosis can cause neuron damage and death, worsening neuroinflammation (50). The effect of Lcn-2 on brain cell apoptosis is controversial. According to Lee et al., Lcn-2 secreted by astrocytes can trigger neuronal death by activating Bim (51). Another study discovered that iron-lacking Lcn-2 binds to 24p3R, is internalized, binds to the intracellular iron carrier, and chelates iron to transfer it outside the cell, thereby reducing intracellular iron levels and inducing the expression of BIM, eventually leading to apoptosis (40). Furthermore, Chen et al. showed that Lcn-2 can also directly initiate neuronal death via mitochondrial apoptotic pathways (52). However, Xing et al. considered that high levels of Lcn-2 can function as a “help-me” signal, which makes glial cells more protective by altering their phenotype that protects neurons in stroke and cerebral ischemia models (53). The role of Lcn-2 on brain cell apoptosis remains to be explored.

### 3.5 Oxidative stress

Oxidative stress refers to the imbalance of redox balance caused by excessive production or insufficient clearance of free radicals such as reactive oxygen species (ROS) which promote neuroinflammation (54). Lcn-2 may affect cell oxidation status by interacting with oxidative stress-related signaling molecules, thereby regulating inflammatory responses. For instance, in a non-alcoholic fatty liver disease (NAFLD) model study, it was found that Lcn-2 could trigger the secretion of HMGB1, activate TLR4 signaling pathway, induce NOX-2 expression, increase ROS production, then cause neuronal oxidative damage (55), which increase neuroinflammation. In addition, a recent study found that mild oxidative stress induced by sodium arsenite reduced the expression of Lcn-2 in cortical astrocytes, which appears to be related to the antioxidant response mediated by the nuclear factor erythroid-2-related factor 2-Kelch-like ECH-associated protein 1 pathway (56). Lcn-2 is involved in the regulation of neuroinflammation through interacting with oxidative stress-related signaling molecules, which provides a target for the treatment of neuroinflammatory diseases.

### 3.6 The blood-brain barrier

Lcn-2 can also affect the BBB, which is a structure that protects the brain from harmful substances and inflammatory factors. Lcn-2 may have both protective and damaging effects on the BBB. For example, a study showed that Lcn-2 may restore endothelial permeability and zonula occludens-1 (ZO-1) expression after focal cerebral ischemia, maintaining the normal structure and function of the BBB in humans (57). But Mondal et al. found that Lcn-2 may disrupt the integrity and function of the BBB by changing the expression of tight junction proteins Claudin5, ZO-1 and increasing the expression of pro-inflammatory cytokines IL-6 and IL-β in brain endothelial cells in NAFLD mouse model (55). These differences may be due to the different pathophysiological processes and mechanisms involved in each disease model. Additionally, the species used in the studies may also contribute to the divergent results, as the BBB may be differently regulated in mice compared to humans. Finally, the experimental conditions, such as the dose and timing of Lcn-2 administration, may also affect the results.

### 3.7 Astrocyte interaction

Lcn-2 can also regulate inflammation by interacting with astrocytes. Some studies have shown that Lcn-2 can promote the activation state of astrocytes, thereby affecting cytokine secretion and neuroinflammation propagation (58). More details will be discussed in detail later.

## 4 Interaction between astrocytes and Lcn-2 in neuroinflammation

In various neurological diseases and neuroinflammation stimuli, astrocytes are activated into reactive astrocytes with A1 and A2 phenotypes, where A1-type astrocytes show harmful effects on neurons by upregulating pro-inflammatory mediators such as ROS, IL-β, IL-6 and TNF-α, while A2-type astrocytes show protective effects by upregulating the expression of neurotrophic factors including glial cell line-derived neurotrophic factor and brain-derived neurotrophic factor that promote neuronal survival and regeneration (16, 17, 59–61). However, recent studies have shown that the phenotype of reactive astrocytes is diverse and dynamic, requiring multidimensional data for classification, rather than simply dividing them into A1 and A2 types (9).

As one of the main sources of Lcn-2 in various neuroinflammatory conditions, reactive astrocytes can regulate the secretion of Lcn-2 in various ways, and Lcn-2 can act as a robust marker of pan-reactive astrocytes and cause changes in them. There is an interactive relationship between Lcn-2 and astrocytes (33, 62). The changes in Lcn-2 levels and astrocytes will profoundly affect the development of neuroinflammation. Thus, understanding their function and activation mechanism will help to find new therapeutic targets and strategies for various neurological diseases.

### 4.1 The mechanism of astrocyte regulation of Lcn-2

**Figure 1:**
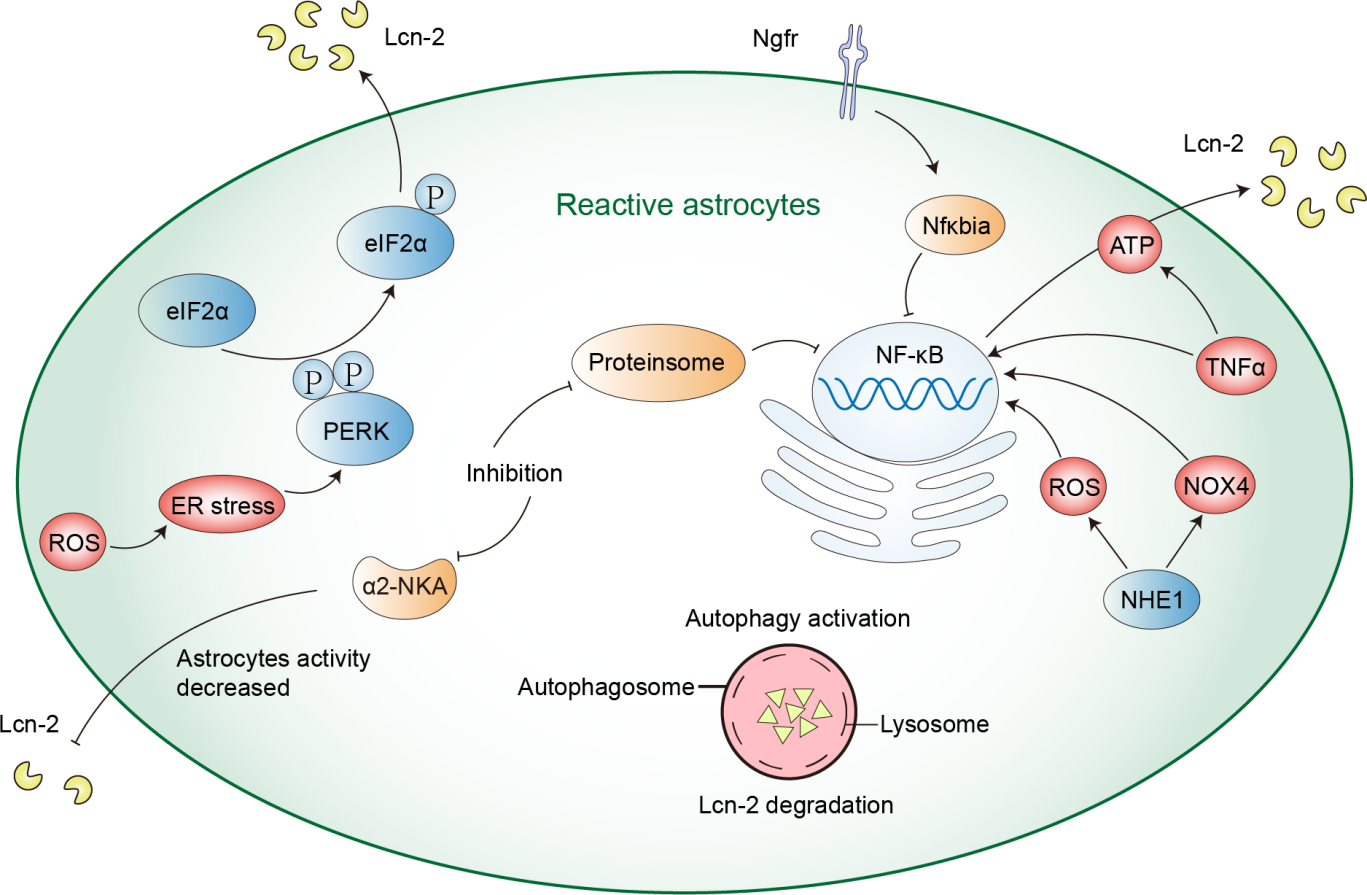
The mechanism of astrocyte regulation of Lcn-2. ROS production can cause ER stress and activate PERK-eIF2α signaling pathway, thus promoting the expression and secretion of Lcn-2. NHE1 protein and TNFα can activate the NF-κB signaling pathway in astrocytes, inducing the secretion of Lcn-2. Inhibiting α2-NKA and proteasome can reduce Lcn-2 secretion by inhibiting the NF-κB signaling pathway, while autophagy activation can reduce its secretion by enhancing intracellular Lcn-2 degradation.

Astrocytes, essential cells in the CNS, can secrete Lcn-2 during neuroinflammatory processes. Besides, stimuli such as ischemia, hypoxia, the Aβ oligomer (AβO), glutamate, etc. can induce astrocytes to generate and release Lcn-2, resulting in neurotoxicity (45, 63, 64). The expression of Lcn-2 is regulated by various signaling pathways, including those involving endoplasmic reticulum stress, ROS generation, the NF-κB activation, among others (52, 65). Here are some mechanisms of the secretion of Lcn-2 regulation in astrocytes (**Figure 1**).

Firstly, the PRKR-like ER kinase-eukaryotic translation initiation factor 2α (PERK-eIF2α) signaling pathway is a mechanism that enables cells to respond to endoplasmic reticulum (ER) stress. This pathway mediates the unfolded protein response (UPR) activated by the accumulation of misfolded proteins in the ER. When astrocytes are activated by UPR, they can upregulate Lcn-2 and reduce synaptic factors *in vivo*, leading to neuronal loss. Inhibiting astrocytic PERK signaling can reduce the activation state of astrocytes, decrease Lcn-2 expression, and increase synaptic factor release, modulating astrocytic UPR signaling to protect neurons (66). Furthermore, after methamphetamine exposure, researchers demonstrated that ROS production and PERK-eIF2α signaling pathway can regulate the expression of Lcn-2. Silencing Lcn-2 or Lcn-2R can mitigate methamphetamine-induced neuronal death, suggesting that the Lcn-2-Lcn-2R axis is a potential therapeutic target (52). The PERK-eIF2α pathway is an important pathway for astrocytes to regulate the secretion of Lcn-2. Secondly, a study has shown that nerve growth factor receptor (Ngfr) can suppress the Lcn-2 activity on Slc22a17, downregulate key transcription mediators Stat1 and Irf8 and upregulate Nfκbia to block NF-κB signaling to inhibit the expression of Lcn-2, thereby promoting astrocyte proliferation and neurogenesis (52). Thirdly, the α2-Na^+^/K^+^ ATPase (α2-NKA) signaling pathway can also affect the secretion of Lcn-2. Inhibiting α2-NKA reduces astrocyte reactivity, lowers the expression of Lcn-2, and protects neurons from tau-induced damage, thus preventing brain atrophy and inflammation (67). Fourthly, TNFα can activate the NF-κB signaling pathway in astrocytes, inducing the secretion of Lcn-2 and altering the bioenergetic spectrum of astrocytes towards mitochondrial oxidative phosphorylation for ATP production, providing energy for the secretion of Lcn-2. Teriflunomide, a drug inhibiting the mitochondrial enzyme dihydroorotate dehydrogenase, shifts astrocyte metabolism towards glycolysis, reducing the secretion of Lcn-2 by limiting ATP production. This action reduces astrocyte inflammatory response by inhibiting the p38 mitogen-activated protein kinase (MAPK) signaling pathway rather than the NF-κB pathway (68). Fifth, astrocyte-specific deletion or inhibition of the Na^+^/H^+^ exchanger1 (NHE1) protein reduces the expression of Lcn-2 and secretion after ischemia, alleviating neuronal damage. NHE1 protein activation triggers NOX4 and ROS production, promoting the NF-κB signaling pathway, thereby regulating Lcn-2 transcription and expression. NHE1 protein represents a potential target for mitigating Lcn-2-mediated neurotoxicity after ischemic stroke (69).

In addition, reactive astrocytes can reduce the secretion of Lcn-2 in two ways: proteasome inhibition and autophagy activation. Proteasome inhibition reduces Lcn-2 transcriptional expression by inhibiting the NF-κB signaling pathway, thereby decreasing its synthesis. Meanwhile, autophagy activation reduces its secretion by enhancing intracellular Lcn-2 degradation, offering protection to neurons. Among them, autophagy flux is negatively regulated by the MTOR signaling pathway and an N-terminal signal peptide is the key to Lcn-2 degradation and secretion (70). In Frontotemporal dementia type 3, Chandrasekaran et al. found that impaired astrocytic autophagy leads to autophagosome accumulation, and reduced autophagy clearance, and affects mitochondrial function and dynamics, this results in reduced astrocyte metabolic activity, increased the secretion of Lcn-2, activated neuroinflammation, and inhibited axonal growth (71). Astrocytic autophagy is essential for Lcn-2 regulation.

Astrocyte-specific deletion or inhibition of proteins such as PERK-eIF2αα2-NKA and NHE1 can reduce the expression and secretion of Lcn-2, and also affect the secretion of Lcn-2 by autophagy, thereby protecting neurons from damage. Astrocytes may also regulate the level of Lcn-2 through other mechanisms, which need further research.

### 4.2 Effect of Lcn-2 on astrocytes

Astrocytes respond to various pathogenic stimuli through reactive programs, which include increased glial fibrillary acidic proteins (GFAP), cell hypertrophy, and profound changes in the secretion of inflammatory factors (72, 73). Reactive astrocytes are crucial contributors to neuroinflammation, releasing inflammatory cytokines and chemokines, and increasing the BBB permeability and immune cell infiltration (18, 19). Upon binding to glial cells, Lcn-2 can activate various inflammatory pathways in glial cells, leading to increased glial cell activation, cytokine production and inflammation exacerbation (20).

**Figure 2:**
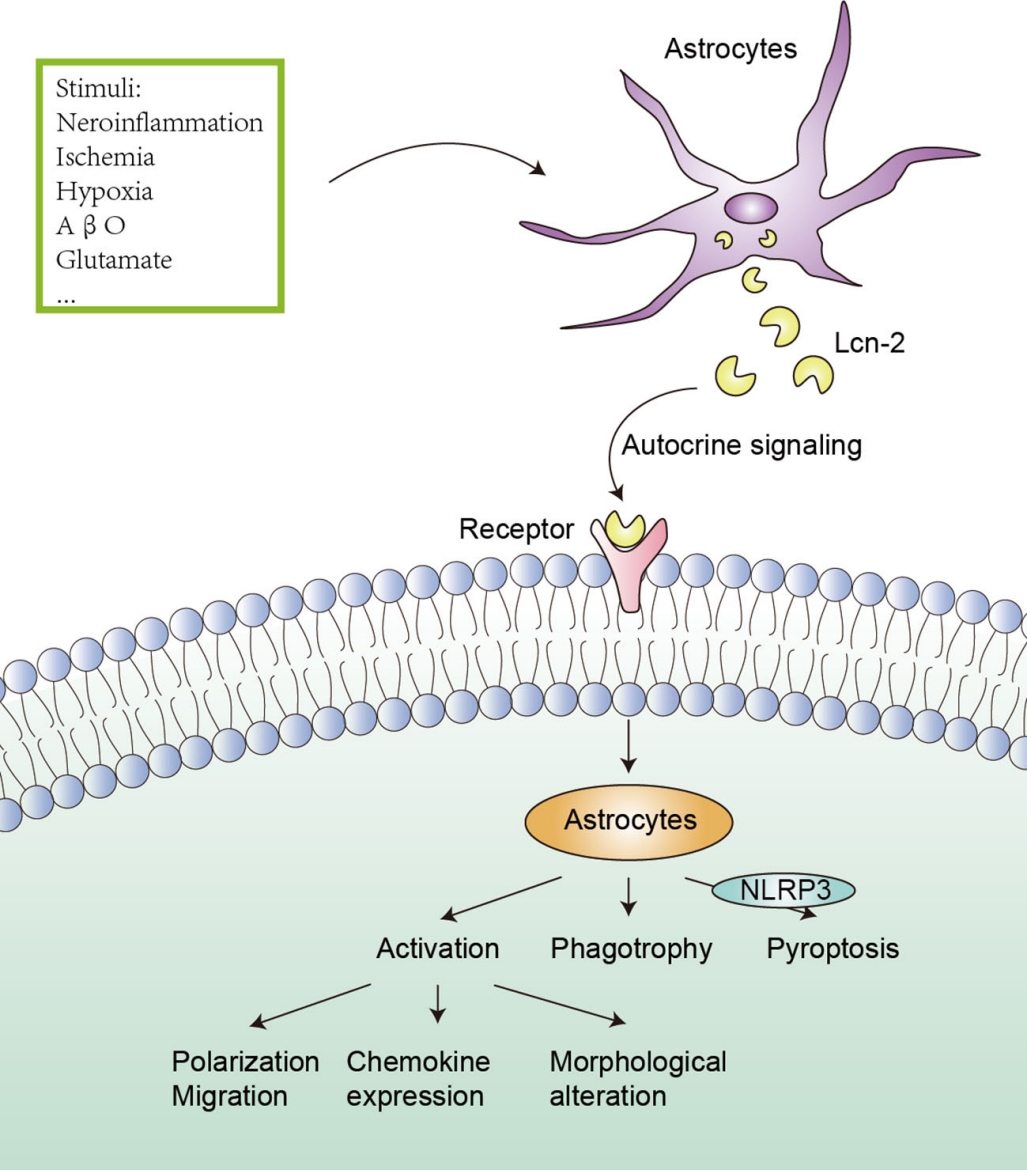
Effect of Lcn-2 on astrocytes. Under the stimulation of neuroinflammation, ischemia, hypoxia, AβO, and glutamate, astrocytes can up-regulate the expression and secretion of Lcn-2, and Lcn-2 can act as an autocrine signal to induce activation (including polarization, migration, chemokine expression and morphological alteration), phagotrophy and pyroptosis (the NLRP3 inflammasome is involved) of astrocytes.

As a multifunctional protein, Lcn-2 can regulate the migration, polarization and activation of astrocytes in the CNS (**Figure 2**). In an experiment to reduce inflammation in zebrafish, Lee et al. discovered that Lcn-2 induces changes in the phenotype of spinal cord astrocytes through the Rho-ROCK (Rho kinase)-GFAP pathway, which is positively regulated by nitric oxide and cGMP. Inhibiting ROCK partially blocks the morphological changes induced by Lcn-2 and the expression of GFAP (74). Moreover, Zhang et al. proved that CXCL10 upregulated by the JAK2/STAT3 pathway in astrocytes is crucial for Lcn-2-induced cell migration in cerebral ischemia (44). Traditionally, Lcn-2 has been viewed as a neurotoxic factor that promotes the shift of astrocytes from an anti-inflammatory to a pro-inflammatory phenotype, characterized by increased chemokine expression and morphological alterations (75). However, an *in vitro* study by Gasterich et al. suggested that the migration, morphology and proliferation of astrocytes can occur independently of Lcn-2 (76). The different results can be influenced by several factors, such as the source of astrocytes, the timing of treatment, and the duration of treatment. Further experiments are needed to validate this.

Lcn-2 affects astrocytes in various neuroinflammatory and neurodegenerative diseases. After oxygen-glucose deprivation, it was found that Lcn-2 overexpression could promote the classical activation pathway of astrocytes in the ischemic hemisphere cortex and hippocampus, manifested by increased iNOS. Lcn-2 exerts adverse effects on astrocyte polarization in ischemic stroke, manifested by increased infarct volume and impaired neurological function. Although the specific mechanism remains unclear, the effect may be mediated by the interaction between Lcn-2 and the 24p3R on astrocytes (23). In another study, Wang et al. demonstrated that Lcn-2/JAK2-STAT3 crosstalk was involved in the activation of neurotoxic microglia and astrocytes in the spinal cord after SCI (47). Across these diseases, elevated levels of Lcn-2 are associated with astrocyte activation. Moreover, Lcn-2 may indirectly regulate the activation of NLRP3 inflammasome through the NF-κB signaling pathway, Lcn-2^−/−^ reduced astrocyte NLRP3 activation in the spinal cord, subsequently decreasing astrocyte proliferation (77). Another study revealed that astrocytic Lcn-2 binds to the 24p3R, activating the NLRP3 inflammasome, and inducing astrocyte pyroptosis and pro-inflammatory factor release in the peri-infarct area after stroke. This cascade of events leads to neuronal death, iron accumulation and BBB disruption, exacerbating neuroinflammation (42). An AD model study also found that neuronal adenosine receptor 1 (A1R) promoted the release of Lcn-2, leading to abnormal activation of astrocytes in the hippocampus, while silencing neuronal Lcn-2 improved astrocyte activation, restored synaptic plasticity and learning/memory. Importantly, the upregulation of A1R is tau pathology-dependent and is modulated by miR-133a-3p-mediated transcription of Mef2c (58). Therefore, Lcn-2 can act as both an autocrine and paracrine factor for astrocytes, activating them by binding to its receptor.

Lcn-2 can also induce astrocytes to phagocytose molecules such as iron and myelin phospholipids, resulting in iron accumulation and demyelination. At the transplant site, astrocyte Lcn-2 interacts with its receptor BOCT, leading to iron deficiency and apoptosis of transplanted neurons (78). Following ischemic stroke, reactive astrocytes in nonischemic areas of the corpus callosum upon injury express Lcn-2 and acquire a phagocytic phenotype capable of uptaking myelin phospholipids. Lcn-2 regulates the phagocytic signaling pathway of astrocytes by binding to low-density lipoprotein receptor-related protein 1 in astrocytes (79). Additionally, Lcn-2 can also trigger activated astrocytes that are sensitive to death (80).

However, it’s important to note that the effects of Lcn-2 on astrocytes are not consistently detrimental. For example, in J20 Alzheimer’s disease mouse model, Lcn-2 did not significantly impact glial cell activation (81). The impact of Lcn-2 on astrocytes appears to vary depending on different disease models, time points and levels, as well as other factors such as the source and signaling pathway of Lcn-2. Consequently, the effects of Lcn-2 on astrocytes may be bidirectional, and further research is needed to elucidate the specific mechanisms and regulatory factors governing the role of Lcn-2 in different neuroinflammatory and neurodegenerative diseases.

Astrocytes become activated and secrete large amounts of Lcn-2 when stimulated. Lcn-2 exacerbates neuronal damage and death through NF-κB signaling pathways and other signaling pathways and mechanisms. At the same time, Lcn-2 can also affect the function of astrocytes, inducing them to produce more inflammatory factors, chemokines and necroptosis factors, thereby further aggravating neuroinflammation. On the other hand, astrocytes can also regulate the level and activity of Lcn-2 by phagocytosis and other factors, forming a negative feedback regulation mechanism. Therefore, there is a complex interaction relationship between Lcn-2 and astrocytes, affecting the development and outcome of neuroinflammation. However, the specific signaling pathways and mechanisms still need further in-depth research to gain a more detailed understanding. In addition, the role of Lcn-2 in neuroinflammation may vary depending on stimulus conditions, cell types and other factors, necessitating verification and comparison under different experimental models and conditions (81–85).

## 5 Lcn-2 and other brain cells

In conditions of neuroinflammation, Lcn-2 is secreted not only by astrocytes but also by other brain cells such as microglia, neurons, and endothelial cells (**Table 1**). Microglia, as the resident macrophages of the CNS, constitute 5% to 15% of adult brain cells and play a crucial role in maintaining immune defense (92). However, uncontrolled activation of microglia can lead to neuroinflammatory responses and neuronal death (93). Additionally, the TNFα and IL-1α secreted by activated microglia can induce astrocytes to adopt the A1 phenotype, thereby amplifying neuroinflammation (17). In the context of neuroinflammation, activated microglia can polarize into either the M1 pro-inflammatory phenotype or the M2 anti-inflammatory phenotype. M1 microglia produce pro-inflammatory mediators, leading to tissue inflammation, which may further contribute to neurodegenerative diseases. Conversely, M2 microglia contribute to mitigating neuroinflammation (94). Research has revealed that Lcn-2, acting as a neuronal signal, induces the formation of pro-inflammatory microglial. In a mouse model of surgery-induced neuroinflammation, knocking down Lcn-2 prevented microglial transition to a pro-inflammatory state and mitigated neuroinflammation (95). Additionally, Wei et al. suggested that Lcn-2 may regulate hippocampal microglial activation via the P38 MAPK pathway within the poststroke depression, polarizing them toward the M1 phenotype (96). However, the role of Lcn-2 in brain ischemia models yields conflicting results. While a study proposes that elevated Lcn-2 levels may shift glial cells toward a protective phenotype, this outcome may be influenced by experimental models and other factors (53). Furthermore, Lcn-2 can mediate apoptosis in microglia. For instance, in methamphetamine-induced striatal microglial apoptosis, the CCAAT-enhancer binding protein/Lcn-2 axis plays a critical role (97).

**Table 1:** Neuroinflammatory disease and main brain cells associated with the secretion of Lcn-2.

Neuroinflammation-related disease	Main brain cells	Reference
Multiple sclerosis	Astrocytes, Microglia	(37)
Parkinson’s disease	Astrocytes	(86)
Alzheimer’s disease	Astrocytes	(45)
Spinal cord injury	Astrocytes, Endothelial cells, Microglia	(47, 65)
Traumatic brain injury	Astrocytes	(87)
Ischemic stroke	Astrocytes, Neutrophils, Endothelial cells	(88)
Hemorrhagic stroke	Astrocytes, Microglia, Macrophages, Neutrophils, Endothelial cells	(89)
Non-alcoholic fatty liver disease	Astrocytes, Microglia, Endothelial cells	(55)
Diabetic encephalopathy	Astrocytes, Microglia	(90)
Post-traumatic stress disorder	Astrocytes	(91)

In addition to astrocytes and microglia, Lcn-2 also impacts other brain cells. It can promote neuronal apoptosis, but it exhibits protective effects in brain ischemia models (40, 53). Additionally, Lcn-2 regulates endothelial cell permeability and inflammatory responses, affecting the BBB integrity and function (57). It also inhibits oligodendrocyte proliferation and differentiation and reduces myelin sheath formation by activating SLC22A17/early growth response protein 1 signaling pathway (98). However, Gasterich et al. believed that Lcn-2 can attenuate oligodendrocyte loss in mouse models for MS (37). Lcn-2 participates in neuroinflammation and the development of neurological diseases by influencing various brain cell types, highlighting its potential as a therapeutic target for these conditions. While the effects of Lcn-2 on brain cells are still not fully understood, further research is needed to explore the roles and potential mechanisms of Lcn-2 in different diseases.

## 6 Lcn-2 from the periphery

Lcn-2 is expressed in several tissues including kidney, lung, bone marrow, liver and others (28). Some studies have shown that Lcn-2 secreted by peripheral organs can enter the CNS through blood circulation and participate in the development of neuroinflammation. For instance, in a NAFLD model, high levels of Lcn-2 in the blood increased the expression of the Lcn-2 receptor (24p3R) on brain cells and stimulated the release of HMGB1. Then induced oxidative stress and activated the NF-κB signaling pathway by triggering the nuclear translocation of p65 protein via NOX-2. Moreover, HMGB1 also activated the NLRP3 inflammasome, which enhanced the secretion of inflammatory cytokines IL-6 and IL-1β from brain cells. These researchers also discovered that high Lcn-2 in the blood disrupted the function of the BBB, mainly by lowering the expression of tight junction protein Claudin5 and elevating the expression of inflammatory cytokines in brain endothelial cells (55). In addition, some researchers considered that Lcn-2 can enter the brain through the cerebrospinal fluid circulation, causing neuroinflammation. However, the mechanism of how Lcn-2 enters the cerebrospinal fluid is unclear (99). It can be seen that peripheral Lcn-2 also effects neuroinflammation.

## 7 The methods of Lcn-2 and astrocytes as targets for neuroinflammation treatment

Lcn-2 is a neuroinflammation regulator that is produced by reactive astrocytes and activates some pathways, leading to neuroinflammation and neuronal death. By inhibiting Lcn-2 production or function, it may be possible to reduce secondary damage and improve neurological outcomes. In addition, Lcn-2 can also inhibit the development of inflammation by reducing the formation of reactive astrocytes and their cytotoxicity. Therefore, Lcn-2 and astrocytes have a potential role in the treatment of neurodegenerative diseases. Its multiple mechanisms of action may provide possibilities for developing new therapeutic strategies.

There are currently many therapeutic methods targeting Lcn-2, which have shown effects in animal models. For example, in the cerebral ischemic injury model, Deng et al. use microRNA-138-5p-overexpressing bone marrow-derived mesenchymal stem cells (100), Sailuotong capsules (44), Lcn-2 monoclonal antibody (88) and voluntary running (101) to reduce the expression or activity of Lcn-2, thereby inhibiting neuroinflammation and neuronal apoptosis, and improving motor function. In the SCI model, Vismara et al. use Rolipram (102), *in situ* Lcn-2 pRNA-RNAi nanotherapy combined with iNSC transplantation (103) and photobiomodulation (47) to reduce the release or upregulation of Lcn-2, thereby limiting the pro-inflammatory phenotype of astrocytes and promoting spinal cord injury repair. In some other nervous system models, Dekens et al. also use iron chelators (104), desferrioxamine (86) and low-intensity pulsed ultrasound (105) to interfere with the signaling pathway or metabolism of Lcn-2, thereby reducing the occurrence of neuroinflammation. Besides, Chen et al. found that electroacupuncture stimulation at Baihui (GV20) and Dazhui (GV14) acupoints can down-regulate the expression of Lcn-2 in the hippocampal astrocytes or repeated social defeat stress-treated mice (91), which provides us a new idea to explore ways to target Lcn-2 in treating neuroinflammation.

Therefore, the current Lcn-2-targeted treatment methods include: specific inhibitors of Lcn-2 to intervene in the overexpression of Lcn-2 in neuroinflammation, antibody neutralization to reduce its interaction with astrocytes, small molecule compounds that regulate the expression of Lcn-2 or activity, targeted drug delivery methods for Lcn-2, etc., as well as combining drug intervention targeting Lcn-2 with other existing treatment strategies. In addition, the mechanism pathways that mediate the interaction between Lcn-2 and astrocytes may be a potential way to improve neuroinflammation. If we can intervene or inhibit these pathways, we may be able to reduce the adverse effects of Lcn-2 and astrocytes on the nervous system. However, this method also has some risks and challenges. For example, will acting on these pathways affect other normal physiological functions? Will it cause adverse side effects or toxicity? Will it interact with other treatment methods? These questions need to be carefully considered and verified by us. And drug intervention targeting Lcn-2 can also be combined with other existing treatment strategies such as immunomodulatory drugs, cell therapy, antioxidants, and neurodegeneration therapy, etc. This can alleviate neuroinflammation from multiple aspects by utilizing the advantages of different treatment methods to achieve more comprehensive, precise and effective interventions and bring better treatment outcomes for patients. However, the selection and optimization of combined treatment strategies need to be verified by in-depth research and clinical experiments.

## 8 Discussion

In conclusion, Lcn-2, as a crucial regulatory molecule in neuroinflammation, mediates a complex signaling network and holds significant therapeutic potential. Among various neuroinflammatory-related diseases, astrocytes are one of the primary sources of Lcn-2. While regulating Lcn-2 levels, astrocytes are also influenced by feedback from Lcn-2, thereby impacting the development of neuroinflammation. By intervening in astrocytes, the expression, and secretion of Lcn-2, and their interactions, it is possible to effectively suppress the progression of neuroinflammation and yield positive outcomes in the treatment of neurodegenerative diseases.

However, it is worth noting that there is still debate regarding whether Lcn-2 exerts exclusively pro-inflammatory effects, and astrocytes are not the sole brain cells that secrete Lcn-2 in the context of neuroinflammation. Future research should continue to explore the molecular interactions and signaling pathways related to Lcn-2 and neuroinflammation, to better understand its role in neurodegenerative diseases. At the same time, the development of new therapeutic methods and drugs based on Lcn-2, as well as the prospect of applying Lcn-2 to clinical diagnosis and treatment are also worth exploring. Through the efforts of multidisciplinary cooperation, Lcn-2 is expected to become an important target for the treatment of neurodegenerative diseases in the future, bringing better quality of life and treatment outcomes for patients.

## Author contributions

QT: Writing – original draft. CZ: Writing – review & editing. XR: Writing – review & editing. WW: Writing – review & editing. WL: Writing – review & editing. SH: Writing – review & editing. JY: Writing – review & editing. YL: Writing – review & editing, Conceptualization, Supervision. FH: Writing – review & editing, Conceptualization, Funding acquisition, Supervision.
